# Ruthenium based metallopolymer grafted reduced graphene oxide as a new hybrid solar light harvester in polymer solar cells

**DOI:** 10.1038/srep43133

**Published:** 2017-02-22

**Authors:** R. Vinoth, S. Ganesh Babu, Vishal Bharti, V. Gupta, M. Navaneethan, S. Venkataprasad Bhat, C. Muthamizhchelvan, Praveen C. Ramamurthy, Chhavi Sharma, Dinesh K. Aswal, Yasuhiro Hayakawa, B. Neppolian

**Affiliations:** 1 SRM Research Institute, SRM University, Kattankulathur, Kancheepuram 603203 (D.t.), Tamil Nadu, India; 2 Organic and Hybrid Solar Cell Group, National Physical Laboratory, Dr. K. S. Krishnan Marg, New Delhi 110012, India; 3 Research Institute of Electronics, Shizuoka University, 3-5-1 Johoku, Naka-Ku, Hamamatsu, Shizuoka 432-8011, Japan; 4 Center for Materials Science and Nano Devices, Department of Physics, SRM University, Kattankulathur, Kancheepuram 603203 (D.t.), Tamil Nadu, India; 5 Department of Materials Engineering, Indian Institute of Science, Bangalore 560012, India; 6 Ultrafast Optoelectronics and Terahertz Photonics group, Physics of Energy Harvesting Division CSIR-National Physical Laboratory, New Delhi, 110012, India; 7 National Physical Laboratory, Dr. K. S. Krishnan Marg, New Delhi 110012, India

## Abstract

A new class of pyridyl benzimdazole based Ru complex decorated polyaniline assembly (PANI-Ru) was covalently grafted onto reduced graphene oxide sheets (rGO) *via* covalent functionalization approach. The covalent attachment of PANI-Ru with rGO was confirmed from XPS analysis and Raman spectroscopy. The chemical bonding between PANI-Ru and rGO induced the electron transfer from Ru complex to rGO *via* backbone of the conjugated PANI chain. The resultant hybrid metallopolymer assembly was successfully demonstrated as an electron donor in bulk heterojunction polymer solar cells (PSCs). A PSC device fabricated with rGO/PANI-Ru showed an utmost ~6 fold and 2 fold enhancement in open circuit potential (*V*_*oc*_) and short circuit current density (*J*_*sc*_) with respect to the standard device made with PANI-Ru (*i.e.,* without rGO) under the illumination of AM 1.5 G. The excellent electronic properties of rGO significantly improved the electron injection from PANI-Ru to PCBM and in turn the overall performance of the PSC device was enhanced. The ultrafast excited state charge separation and electron transfer role of rGO sheet in hybrid metallopolymer was confirmed from ultrafast spectroscopy measurements. This covalent modification of rGO with metallopolymer assembly may open a new strategy for the development of new hybrid nanomaterials for light harvesting applications.

Ruthenium (Ru) complexes have gained significant interest in energy conversion applications owing to its attractive opto-electronic properties such as good photostability, excellent light harvesting capability and efficient charge transport behaviour^1^,^2^,^3^. Especially, the Ru complexes with benzimidazole ligands have been attracted as a photosensitizer in dye sensitized solar cells^4^. The highly conjugated aromatic rings of benzimidazole complex could efficiently absorb light and thereby promotes the electron to the metal centers^5^. Several strategies have been proposed recently to develop different types of benzimidazole based hybrid metallopolymer assemblies for energy conversion process^6^,^7^. The incorporation of benzimidazole based complexes with conjugated polymers not only provides a long range of π electron network but also facilitates the electron transfer through the conjugated polymer chain as the backbone^5^. It has also been extensively studied that the linkage existing between the dπ orbitals of metal centers and π or π* orbitals of the conjugated polymer induces the electron transfer in metallopolymer systems^5^. Moreover, the functionalization of metal complex with polymer offers high optical absorption, relatively long lived metal to ligand charge transfer (MLCT) and better photo stability^8^. However, the possibility of back flow of charge carriers from the conjugated polymer to metal center greatly affects the implementation of metallopolymer in solar energy conversion applications^9^. Thus, the further attachment of photoactive metallopolymer assembly with highly conductive two-dimensional graphene support not only prevent the back flow of the carriers but also enhance the carrier separation, electron mobility and thereby considered as a superior material for light harvesting applications^10^,^11^.

Graphene is a rising-star of two dimensional material made up of single sheet of hexagonally packed carbon atoms with attractive properties such as large surface area, high intrinsic mobility, better optical transparency and thermal stability^12^,^13^,^14^,^15^,^16^,^17^, which further turn it as an impressive candidate for variety of applications including ultracapacitors, sensors, photocatalysis, actuators, fuel cells and solar cells etc.^18^,^19^,^20^,^21^,^22^,^23^,^24^. The large surface area and active edges associated with the two dimensional reduced graphene oxide (rGO) sheets offer several advantages to incorporate various organic or inorganic moieties to develop hybrid nanocomposites for optoelectronic applications^25^,^26^. In particular, the covalent functionalization of rGO or graphene sheet have attracted numerous attentions in polymer solar cells (PSCs) technology^27^,^28^. For instance, the edge functionalization of graphene oxide (GO) with Cs metal effectively tuned the work function of GO, which further matched with the work function of lowest unoccupied molecular orbital (LUMO) level of PCBM for the effective separation of electrons^29^. The covalently grafted C_60_ fullerene with graphene sheet possessed an improved electron transport in P3HT based PSC and hence the overall power conversion efficiency (PCE) of device was enhanced^30^. Similarly, P3HT polymer was functionalized with the carboxylic acid groups of GO through esterification reaction and the covalent linkage achieved between P3HT and GO significantly improved the PCE^31^. However, most of the work reported earlier contains the modification of existing electron donor and acceptor polymers (P3HT and PCBM) for PSC. The covalent functionalization of graphene with light harvesting metallopolymer assembly can combine the interesting photophysical properties of metallopolymers with the excellent electronic properties of graphene. Therefore, the strategy to design graphene-metallopolymer nanocomposite open up the potential platform for the development of new hybrid nanomaterials as an electron donor/acceptor for PSCs. In addition, the loading of graphene can enhance the mechanical stability of the metallopolymer moiety which is an additional benefit of using graphene as a supporting material^32^.

Our interest is to develop hybrid graphene-metallopolymer assembly as an electron donor for PSC and investigate the carrier separation and electron transport properties of graphene along with metallopolymer. Herein, pyridyl benzimidazole liganded Ru complex was bonded with polyaniline (PANI) and this metallopolymer (PANI-Ru) assembly was further covalently grafted on rGO sheets through simple chemical approach. This is the first report of using {2-(2-pyridyl)benzimidazole}RuCl_3_ based metallopolymer functionalized rGO sheets as a new class of hybrid light harvester (electron donor) for PSC. The interfacial electron transport and charge separation properties of rGO at the metallopolymer (PANI-Ru) donor/PCBM acceptor interfaces significantly improved both short circuit current density (*J*_*sc*_) and open circuit potential (*V*_*oc*_) of the PSC device.

## Results and Discussion

**Figure 1:**
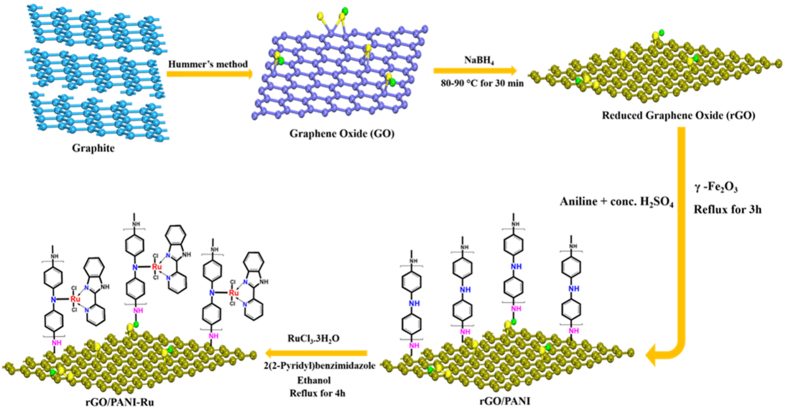
Synthesis procedure of rGO/PANI-Ru hybrid nanocomposite.

The synthesis procedure of rGO/PANI-Ru hybrid nanocomposite is illustrated in Fig. 1 (*synthesis procedure was described detail in the experimental methods*). Initially, graphene oxide (GO) was synthesized from graphite powder using modified Hummer’s method. Then, GO was reduced into rGO using NaBH_4_ as a reducing agent. The PANI is covalently grafted on rGO sheet in the presence of aniline monomer, conc.H_2_SO_4_ and γ-Fe_2_O_3_. Finally, the functionalization of {2-(2-pyridyl)benzimidazole}RuCl_3_ complex on PANI is achieved under reflux condition.

**Figure 2:**
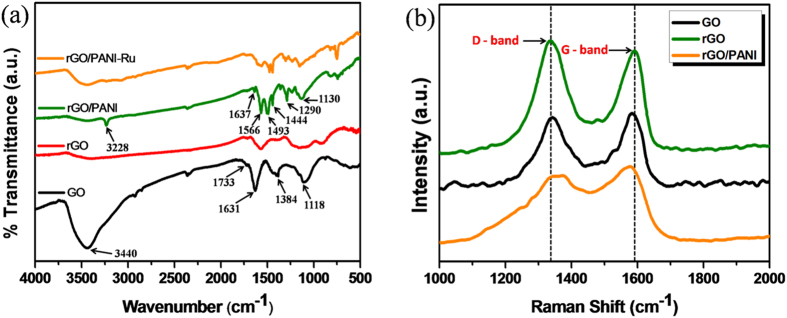
(**a**) FT-IR spectra of GO, rGO, rGO/PANI and rGO/PANI-Ru and (**b**) Raman spectra of GO, rGO and rGO/PANI.

The linkage between metallopolymer (PANI-Ru) and rGO was confirmed through FTIR and Raman spectra (Fig. 2). The FTIR spectra of GO, rGO, rGO-PANI and rGO/PANI-Ru are displayed in Fig. 2a. The GO exhibits a broad absorption band at ~3440 cm^−1^ is assigned to the stretching vibration of hydroxyl groups^33^. The absorption peaks located at 1631 and 1384 cm^−1^ are associated with stretching vibration of aromatic C=C bond and bending vibration of O–H groups, respectively^34^. The additional absorption peaks at 1733 and 1118 cm^−1^ are related to the stretching vibrations of C–O and C=O groups, respectively^35^. In case of rGO, the peak intensities of oxygen rich functional groups and hydroxyl groups are significantly reduced compared to that of GO, which further confirmed the reduction of GO to rGO^36^. Similar FTIR spectra were also reported in the literatures for rGO^36^,^37^,^38^. The rGO/PANI nanocomposite exhibits several absorption peaks other than rGO. More importantly, three peaks observed at 1637, 1444 and 1290 cm^−1^ are assigned to the stretching vibrations of C=N, C=C and C–N bonds, respectively^36^. An additional peak occurred at 3228 cm^−1^ corresponds to the N–H stretching vibration of PANI in rGO/PANI. Besides, two more peaks appeared at 1566 and 1493 cm^−1^ are attributed to the bending vibration of C–H groups in quinonoid ring and stretching vibration of C=C bonds in benzonoid rings, respectively^39^. It is worth mention to note that a peak identified at 1130 cm^−1^ corresponds to stretching vibration of N–Q–N–Q bonds from the quinonoid rings, which further suggesting the covalent grafting of PANI on rGO sheets. For rGO/PANI-Ru, the disappearance of N–H peak clearly indicates the successful bond formation between Ru complex and PANI. Furthermore, the rGO/PANI-Ru hybrids exhibit few more new absorption bands compare to rGO/PANI, which might be due to the presence of {2-(2-pyridyl) benzimidazole}RuCl_3_ complex in the resultant nanocomposites. Several absorption bands observed for pure PANI and Ru complex were represented in Fig. S1 (Supplementary Information).

The reduction of GO and the covalent grafting of PANI on rGO sheets were further confirmed through Raman spectra. As shown in Fig. 2b, the Raman spectra of GO displays two characteristic Raman bands centered at 1342 at 1581 cm^−1^ are assigned to the well known D and G bands, respectively. In general, the D band is known as defective band that usually arises from the first order scattering of sp^3^ hybridized carbon atoms, while the G band is mainly reflected from the stretching vibration of sp^2^ hybridized C=C bonds^40^. During the reduction of GO to rGO, the defects on the surface of the rGO increases which relatively increase the D band intensity compare to that of GO, as shown in Fig. 2b. The calculated *I*_*D*_*/I*_*G*_ ratio value of GO and rGO are 0.95 and 1.06, respectively. Thus, the increase in *I*_*D*_*/I*_*G*_ value of rGO clearly attributes the formation of disordered new graphitic domains during the reduction process^41^. When PANI was grafted with rGO, a notable shift in G band is observed towards lower wavenumber region from 1591 cm^−1^ of rGO to 1577 cm^−1^ indicates the chemical bond formation between rGO sheet and PANI chain^42^. More interestingly, the *I*_*D*_*/I*_*G*_ value of rGO/PANI is decreased to 0.79, which is less than the calculated *I*_*D*_*/I*_*G*_ value of rGO. This clearly reveals that the grafting of PANI was occurred on the defective sites of the rGO, which further repaired the defects and restored the basal plane conjugation with minimal defects on the surface of rGO^43^.

**Figure 3:**
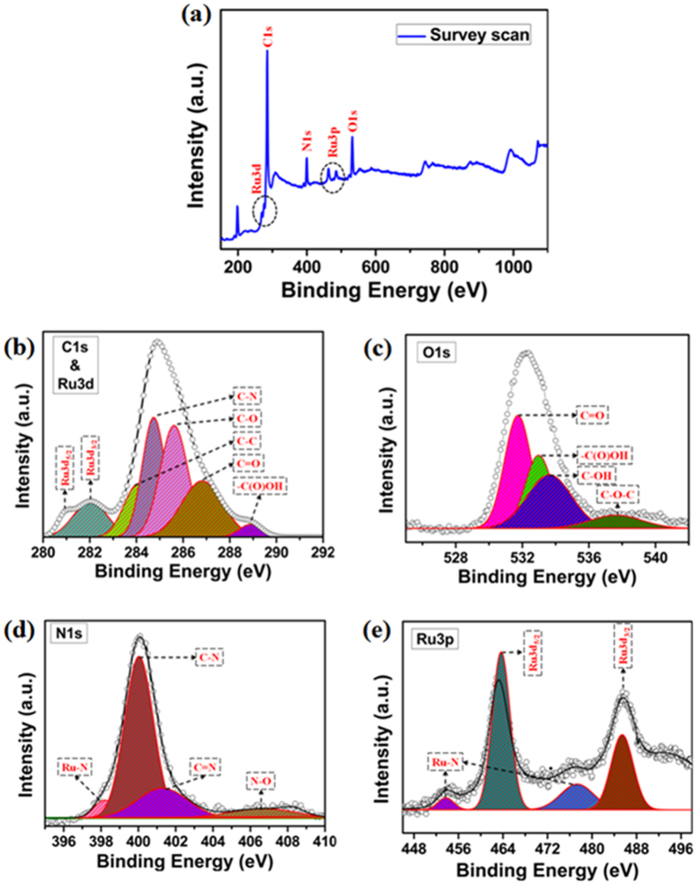
XPS spectra of rGO/PANI-Ru hybrid nanocomposite (**a**) wide angle, (**b**) high resolution C 1 s and Ru 3d, (**c**) high resolution O 1 s, (**d**) high resolution N 1 s and (**e**) high resolution Ru 3p spectra.

X-ray photoelectron spectroscopy (XPS) analysis was performed to understand the composition, chemical oxidation state of metal (Ru) and also to know the chemical environment of the elements present in the rGO/PANI-Ru composite sample, as depicted in Fig. 3. As can be seen from Fig. 3a, the Ru3d, C1s, N1s, Ru3p and O1s peaks are observed from the wide angle spectrum of rGO/PANI-Ru catalyst. The peaks centered at 285 eV, 534 eV and 401 eV correspond to the elemental composition of C1s, O1s and N1s^44^,^45^. Besides, the Ru3d and Ru3p bands are identified at 280.9–282.1 eV and 453.7–485.4 eV, respectively^46^.

The binding energy values of Ru3d and C1s are fairly close and hence the bands are observed with the binding energy difference of ~3 eV, as evident from the high resolution C1s and Ru3d spectrum given in Fig. 3b 47. Two Ru3d species observed at 281.0 eV and 282.0 eV correspond to Ru3d_5/2_ and Ru3d_3/2_, respectively. As shown in Fig. 3b, the high resolution C1s spectrum is deconvoluted into five different components *i.e.,* C–C, C–N, C–O, C=O and –COOH^23^. The three functional groups such as C–O, C=O and –COOH are emerges from the rGO. The peak located at 284.1 eV is arise from the combination of graphitic C–C bond of rGO along with the aromatic C–C bond from the PANI and the organic ligand of the Ru complex. Likewise, the C–N bond peak appeared at 284.7 eV is might be due to the carbon-nitrogen bond present in PANI and also in Ru complex^48^,^49^,^50^.

As displayed in Fig. 3c, deconvolution of O1s spectrum consists of four different chemical components namely C=O, –COOH, –C–OH and –C–O–C–. The respective binding energy values are noticed at 531.7, 532.9, 533.6, 537.6 eV for the four different oxygen functionalities^51^. These are the residual oxygen functional groups present in rGO after the reduction of GO using NaBH_4_. However these functional group remains present on rGO induces the chemical bond formation with PANI and increased the π–π interaction between PANI and rGO to facilitate the electron transfer^52^.

The C–N peak observed at the high resolution N1s XPS spectrum resulted from the carbon-nitrogen bond present in the 2-(2-pyridyl) benzimidazole ligand of the Ru complex and also present in the PANI. As can be seen from Fig. 3d, the adjacent deconvoluted peak centered at 401.2 eV is evolved from the –C=N bond of the organic ligand in the Ru complex. Very importantly, the peak at 406.6 eV corresponds to the N–O bond. This clearly evident the formation of nitrogen-oxygen bond between PANI and rGO noticeably sustained the formation of chemical interaction that connecting PANI with rGO support in some places^53^. In addition, Ru–N peak observed at 398.1 eV strongly evident the complexation of RuCl_3_ with the 2-(2-pyridyl)benzimidazole ligand.

Alike Ru3p peaks are identified around 460 to 492 eV region. Two distinct peaks noted at 463.7 and 485.4 eV correspond to the Ru3d_5/2_ and Ru3d_3/2_, respectively (Fig. 3e). In addition, two more peaks centered at 453.7 and 485.4 eV are might be owing to the Ru–N bond of the Ru complex^54^. This significantly proven the formation of Ru complex which is obtained from the reaction between the N,N′-bidonor ligand [2-(2-pyridyl)benzimidazole] and RuCl_3_·3H_2_O.

**Figure 4:**
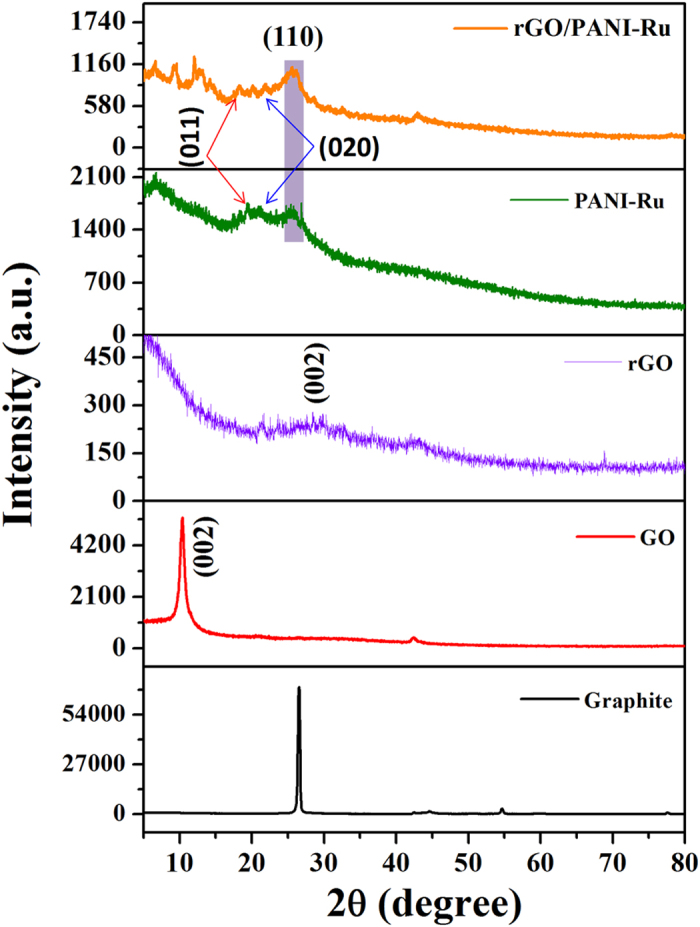
XRD patterns of graphite, GO, rGO, PANI-Ru and rGO/PANI-Ru.

XRD patterns of Graphite, GO, rGO, PANI-Ru and rGO/PANI-Ru composites are illustrated in Fig. 4. It can be seen that the prepared GO exhibits a sharp and intense diffraction peak at 2θ = 10.3° which is related to the (002) crystalline plane^34^. In the case of rGO, a sharp peak at 10.3° is completely disappeared and a new broad peak appears at 2θ = 28.4° clearly suggests that the oxygen rich functionalities were significantly reduced and thereby rGO was formed. The characteristic diffraction peaks of PANI from PANI-Ru and rGO/PANI-Ru nanocomposites are observed at 2θ = 19.4°, 20.9° and 25.4° corresponds to (011), (020) and (110) crystal planes, respectively, which further ascribed the well crystalline nature of PANI^34^,^52^. A small peak centered at 28.4° of resultant hybrids confirmed the existence of rGO. Moreover, a small diffraction peak noted at 2θ = 6.5° of both PANI-Ru and rGO/PANI-Ru nanocomposite indicates that the functionalization of Ru complex with PANI broadened the interlayer spacing of the PANI^52^. In addition to this, few more less intense diffraction peaks occurred at low angle region of rGO/PANI-Ru hybrids clearly implies that the interlayer distance of the resultant nanocomposite was increased, which might be due to the grafting and intercalation of PANI-Ru with rGO sheet. Thus, the observed XRD results are in good agreement with FTIR and Raman results.

**Figure 5:**
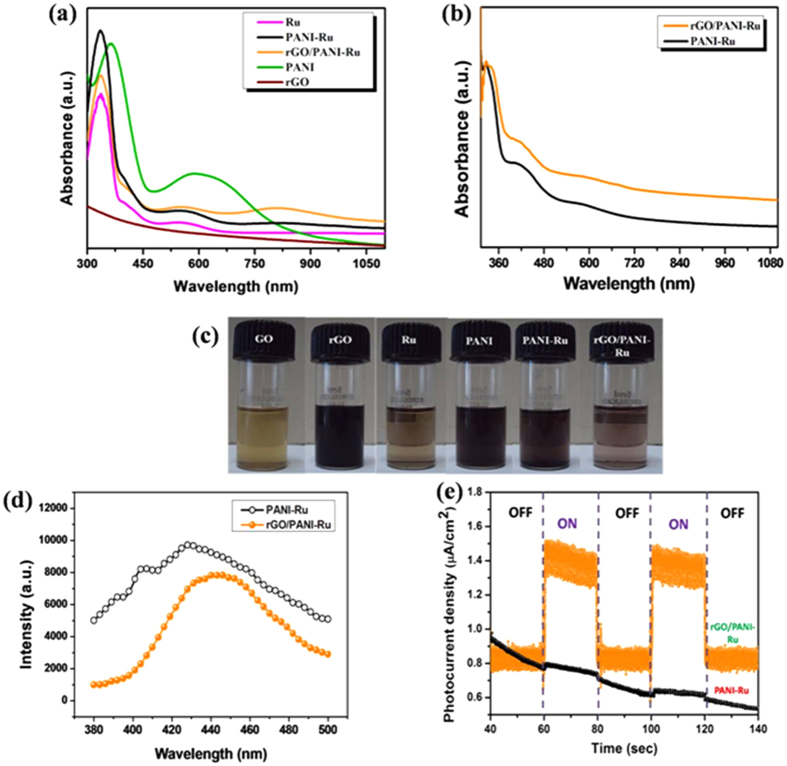
(**a**) UV-visible absorption spectra of Ru, PANI, rGO, PANI-Ru, rGO/PANI-Ru dispersed in DMF solvent, (**b**) Absorption spectra of PANI-Ru and rGO/PANI-Ru films and (**c**) Photograph images of GO, rGO, Ru, PANI, PANI-Ru and rGO/PANI-Ru dispersed in DMF, (**d**) Photoluminescence emission spectra of PANI-Ru and rGO/PANI-Ru nanocomposites and (**e**) Transient photocurrent measurements of PANI-Ru and rGO/PANI-Ru nanocomposites.

Fig. 5a shows the UV-vis absorption spectra of {2-(2-pyridyl)benzimidazole}RuCl_3_ complex, PANI-Ru, rGO/PANI-Ru and rGO dispersed in DMF solution. For prepared PANI, the characteristic absorption peaks noted at 365 nm and 587 nm are emerges from the excitonic transitions of π and π* polarons-benzoid to quinoide, respectively^55^. The pure Ru complex exhibits a strong absorption band at 334 nm which corresponds to π–π* transition of 2(2-pyridyl)benzimidazole ligand^56^. A wide absorption band observed between 460 to 650 nm for Ru complex, PANI-Ru and rGO/PANI-Ru are mainly attributed to a metal to ligand charge transfer (MLCT) between Ru metal and 2(2-pyridyl)benzimidazole ligand^4^. Moreover, a significant blue shift in absorption is noted, when Ru complex is functionalized with PANI. This clearly reveals that the electronic interaction between Ru complex and PANI is the origin for the blue-shift in the absorption compare to that of pure PANI. Furthermore, PANI-Ru grafted with rGO sheet shows an additional broad absorption band from 700 to 900 nm compared to PANI-Ru alone. This is possibly due to charge transfer between rGO sheets and PANI-Ru. As evident from earlier reports, PANI and Ru complexes are fairly good electron donor materials, where as rGO sheet is a good electron acceptor^3^,^39^. During the reflux process aniline could interact with graphene sheet which can easily form graphene-polyaniline composite^39^. Therefore, the observed blue-shift for rGO/PANI-Ru indicates the successful grafting of PANI-Ru over rGO sheet. Moreover, no absorption is noted for rGO dispersed in DMF. Fig. 5b shows the absorption spectra for PANI-Ru and rGO/PANI-Ru films. Both PANI-Ru and rGO/PANI-Ru films exhibit the same characteristic absorption peak at 327 nm and shoulder peaks at 582 and 682 nm. Interestingly, the rGO/PANI-Ru film shows slightly enhanced absorption in the entire solar spectrum compare to that of PANI-Ru alone. The relative absorption increase for rGO/PANI-Ru with respect to that of the PANI-Ru was calculated to be 12.2%. The photograph images of GO, rGO, PANI-Ru and rGO/PANI-Ru dispersed in DMF solution are displayed in Fig. 5c.

To investigate the photo-excited electron transfer in PANI-Ru and rGO/PANI-Ru composites, the photoluminescence spectra (PL) were recorded. Fig. 5d represents the PL emission spectra of PANI-Ru and rGO/PANI-Ru hybrids dispersed in DMF solution excited at 330 nm under room temperature. It can be seen that the PANI-Ru and rGO/PANI-Ru exhibit PL emission bands centered at 427 nm and 440 nm, respectively, which arise from the MLCT Excited state emission. But more interestingly, rGO/PANI-Ru composite shows notable quenching in PL intensity which clearly inferred that rGO in the hybrid composite act as a charge trapping centre for the excited sate MLCT and prevents the charge carrier recombination^42^. In addition, transient photocurrent measurement was also performed to study the charge transfer and recombination process of the excited charge carriers in the hybrid nanocomposites (Fig. 5e). It is interesting to note that the rGO/PANI-Ru shows enhanced and stable photocurrent response in several ON-OFF cycles, whereas negligible photocurrent is obtained for PANI-Ru composites suggesting the potential role of rGO in charge separation.

**Figure 6:**
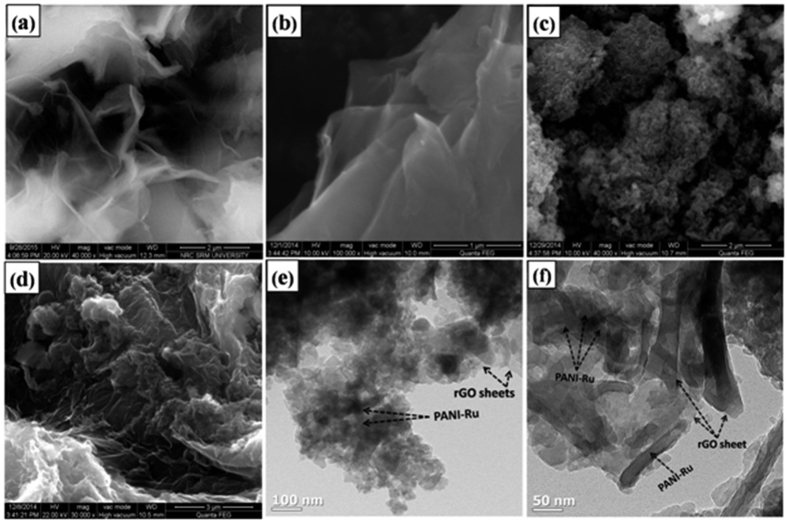
FE-SEM image of (**a**) GO, (**b**) rGO, (**c**) PANI-Ru, (**d**) rGO/PANI-Ru and (**e and f**) TEM image of rGO/PANI-Ru nanocomposite with different magnification.

The morphology of the nanocomposites were analyzed through FE-SEM and TEM analysis. Fig. 6a shows the typical FE-SEM image of synthesized GO, which exhibits the well exfoliated layered 2D morphology with the size of few μm. Similarly, the rGO shows typical 2D sheet like nanostructure with wrinkled surface or edges (Fig. 6b). As shown in Fig. 6c, PANI-Ru exhibits the agglomerated nanostructure. However, the rGO/PANI-Ru shows the 3D nanostructured morphologies due to intercalation and grafting of PANI-Ru onto rGO sheets (Fig. 6d). The EDX spectra clearly confirmed the presence of expected elements such as C, O, N and Ru in rGO/PANI-Ru (Supplementary Information Fig. S2). The FE-SEM image of pure PANI and PANI grafted rGO were displayed in Fig. S3. It was clearly seen that PANI were grafted and intercalated onto the rGO sheet. The grafting of PANI-Ru on rGO was further confirmed through TEM images. Fig. 6e and f displays the typical TEM images of rGO/PANI-Ru with different magnifications. It can be seen that wire nanostructured PANI-Ru is intercalated as well as grafted on the rGO sheets. The TEM image of pristine rGO sheet was represented in Fig. S4 (Supplementary Information).

### Polymer solar cell (PSC) device performance (ITO/PEDOT:PSS/PANI-Ru or rGO/PANI-Ru:PCBM/Al)

**Figure 7:**
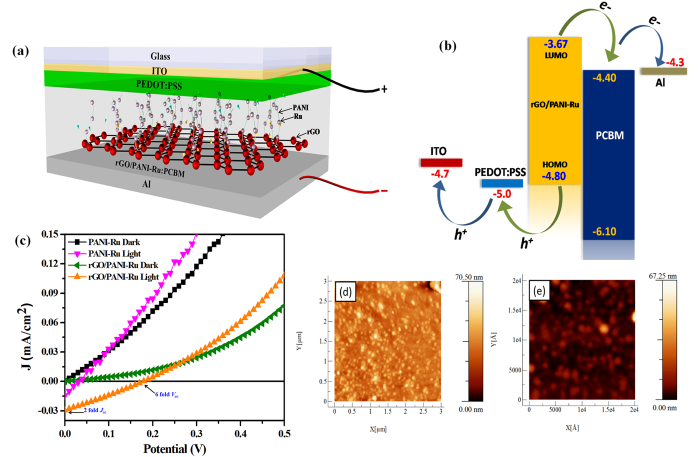
(**a**) Schematic diagram of PSC device fabricated using rGO/PANI-Ru as an electron donor, (**b**) Energy level diagram of rGO/PANI-Ru in PSC, (**c**) current density-voltage (J-V) characteristics of PSC device using PANI-Ru and rGO/PANI-Ru nanocomposites as an electron donor and (**d** and **e**) Tapping mode AFM image of PANI-Ru:PCBM and rGO/PANI-Ru:PCBM films.

To evaluate the photovoltaic behavior of metallopolymer grafted rGO hybrid nanocomposites, bulk heterojunction PSC devices were fabricated with the device configuration of [ITO/PEDOT:PSS/PANI-Ru (rGO/PANI-Ru)PCBM/Al]. The light harvesting PANI-Ru and rGO/PANI-Ru nanocomposites were used as an electron donor (*p*-type) along with fullerene acceptor (PCBM as *n*-type material) as a photoactive layer. Fig. 7a, shows a schematic diagram of PSC device using rGO/PANI-Ru:PCBM as a photoactive layer. The energy level diagram of rGO/PANI-Ru hybrid based PSC is displayed in Fig. 7b. The exact highest occupied molecular orbital (HOMO) and lowest unoccupied molecular orbital (LUMO) energy value of hybrid rGO/PANI-Ru nanocomposite was estimated from cyclic voltammetry (CV) studies. The CV curve for rGO/PANI-Ru was presented in Fig. S5 (Supplementary Information). The HOMO and LUMO value of rGO/PANI-Ru was calculated using the following equation^57^.


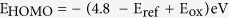



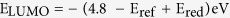



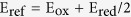


Where E_ox_ and E_red_ are the onset oxidation and onset reduction potential, respectively.

The calculated HOMO and LUMO level of rGO/PANI-Ru are −4.8 eV and −3.67 eV, respectively. Thus, the electrons can easily transfer from rGO/PANI-Ru to PCBM. In addition, the electron transfer from rGO to Al cathode via PCBM acceptor is energetically favourable due to exact matches in the LUMO energy levels between rGO and PCBM^42^. Likewise, the HOMO level work function of rGO/PANI-Ru is close to the work function of PEDOT:PSS for efficient transfer of photogenerated holes. Fig. 7c represents the J-V characteristic curves of PANI-Ru or rGO/PANI-Ru based PSC devices. As can be seen from Fig. 7c, no short circuit current density (*J*_*sc*_) is observed for both PANI-Ru or rGO/PANI-Ru based PSC devices under dark condition. However, *J*_*sc*_ and open circuit potential (*V*_*oc*_) of 0.03 mA/cm^2^ and 0.19 V are achieved for rGO/PANI-Ru based PSC device under the illumination of AM 1.5 G. In contrast, the reference device made with PANI-Ru *i.e.,* without rGO shows less *J*_*sc*_ and *V*_*oc*_ of 0.014 mA/cm^2^ and 0.03 V under identical condition. In the absence of rGO, the charge carriers excited in PANI-Ru nanocomposite undergoes oxidative recombination process and thereby affects the transfer of electrons to PCBM. The achieved *V*_*oc*_ and *J*_*sc*_ of rGO/PANI-Ru used PSC device is 6 fold and 2 fold higher than the device made without rGO (i.e., PANI-Ru alone) under identical experimental conditions. This observation clearly resembled that the rGO in the hybrid nanocomposite not only provides an external electron transfer pathway but also enhances the carrier separation and transportation process to improve the photovoltaic property of hybrid PSC device. Table 2 illustrates the photovoltaic parameters of the PSC device fabricated with PANI-Ru and rGO/PANI-Ru. It is clearly seen that the device made using rGO/PANI-Ru nanocomposite shows ~14.5-fold enhancement in power conversion efficiency (PCE) compared to that of the device fabricated with PANI-Ru. This remarkable improvement in PCE is mainly attributed to the effective separation of charge carriers and ultrafast transportation of electrons in the presence of rGO sheet. Besides, the observed low PCE of the devices might be due to not so well band edge matching of the various layers in the device architecture, limited optical absorption and formation of relatively thin active layer because of the less solubility of rGO/PANI-Ru in *ortho*-dichlorobenzene solvent as reported earlier^42^. Fig. 7d and e shows the AFM images of photoactive film made of PANI-Ru:PCBM and rGO/PANI-Ru:PCBM nanocomposites. The bright spots observed in the AFM images indicate the non-continuous formation of PANI-Ru with aggregated nanostructure^42^.

### Charge Transfer Mechanism

**Figure 8:**
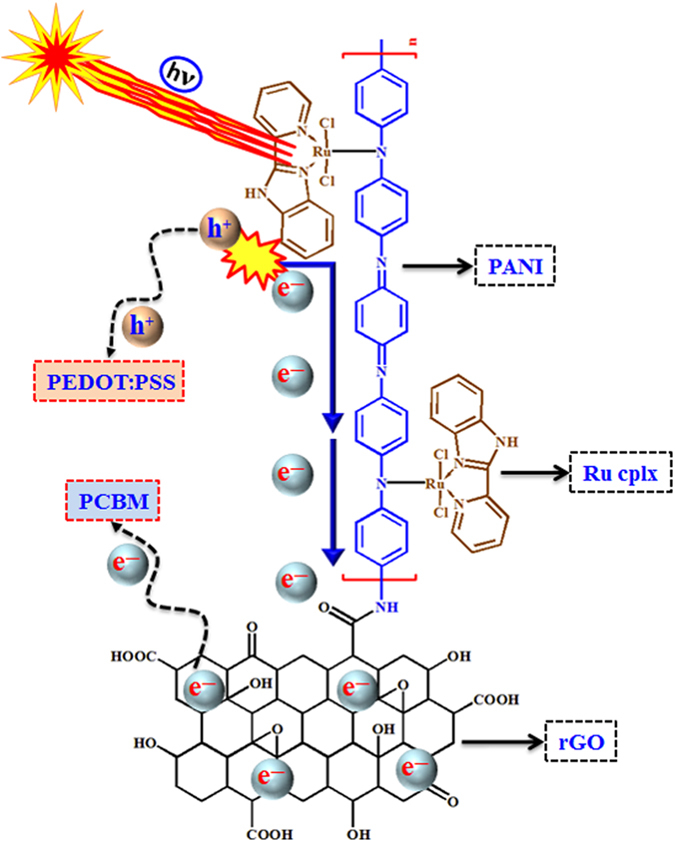
Electron transfer mechanism of rGO/PANI-Ru nanocomposite.

Based on the observation, a detailed electron transfer mechanism involved in rGO/PANI-Ru nanocomposite is illustrated in Fig. 8. In general, Ru-based complexes with aromatic moieties exhibit high light absorbing power^58^. In the present scheme the Ru complex formed with N,N’-bidonor ligand [2-(2-pyridyl)benzimidazole] behaved as a light harvesting antenna. It is worth to mention here is that rGO/PANI-Ru composite exhibited strong light observing capability as robustly manifested from the UV-vis characterization studies. The utmost π—electrons associated in the complex molecule made the system more attractive as an electron source. In addition to that the free electrons present in the N-atom (lone pair of non-bonded electrons) also enhance the electron cloud in the moiety. Once the external light source illuminated on to the system, the electrons (e^−^) are excited to the higher energy states which left positive holes (h^+^) in the Ru complex molecule. These photo-excited electrons migrate to PANI through the Ru—N bond. The presence of Ru—N bond was strongly evident from the XPS results described in Fig. 3. It is a well known fact that PANI is a very good conducting polymer and hence the electrons flow through PANI easily^59^. These high energy electrons further move to the rGO layer. Furthermore these electrons are collected at the Al cathode *via* PCBM and in turn augmented the solar cell performance. Concurrently, the holes are transferred to ITO via by PEDOT:PSS, which further satisfy the electron loss from the Ru complex. The whole process made the Ru-PANI/rGO nanocomposite material more suitable for the solar cell application.

### Ultrafast Spectroscopy Measurements

**Figure 9:**
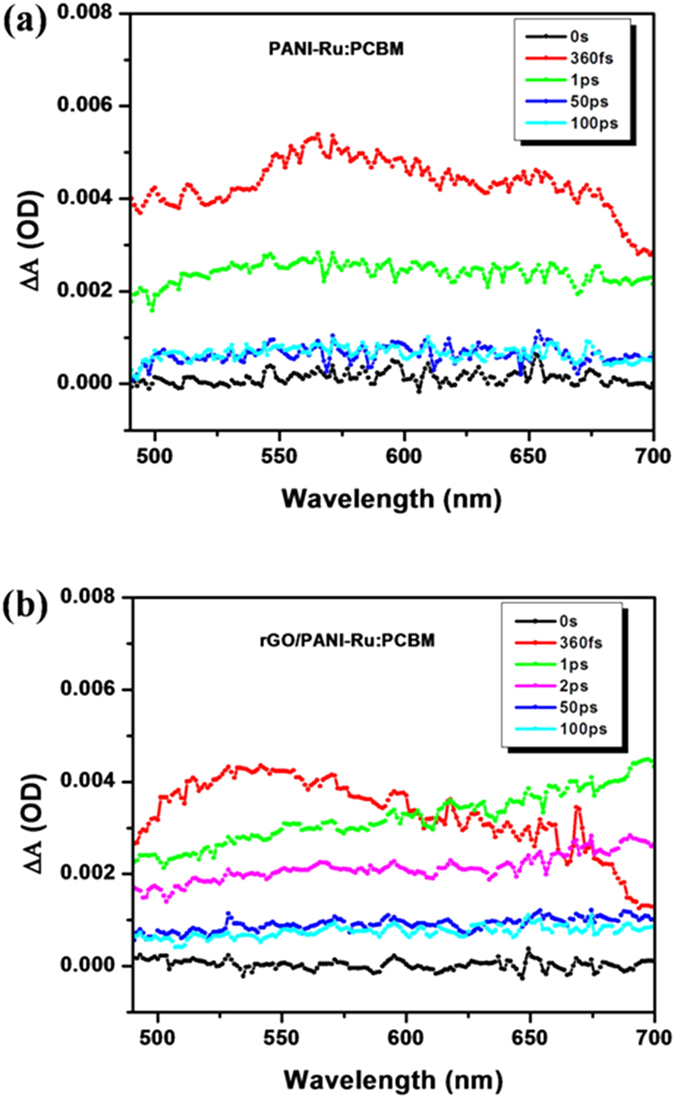
Transient absorption spectra of (**a**) PANI-Ru:PCBM and (**b**) rGO/PANI-Ru:PCBM films.

Ultrafast femtosecond transient absorption spectroscopy (UFTS) was performed by using 330 nm pump and the white light probe (500–700 nm) as a function of time delay between pump and probe pulses. The UFTS measurements were employed to explore the charge transfer role of rGO in hybrid metallopolymer nanocomposites. The proposed charge transfer mechanism involved in metallopolymer grafted rGO hybrid was already described in Fig. 8. The transient absorption (TA) spectra of PANI-Ru:PCBM and rGO/PANI-Ru:PCBM film monitored at ultrafast time scale (few femtoseconds to few nanoseconds) are displayed in Fig. 9. It is clearly observed that both PANI-Ru:PCBM and rGO/PANI-Ru:PCBM photoactive films exhibit a broad excited state absorption (ESA) in the visible range (500 nm-700 nm). The TA spectra of both the films show maximum ESA (at ~560 nm) within ~360 fs. However, Fig. 9b clearly depicts that rGO/PANI-Ru:PCBM, excited state absorption decreased comparatively, showing the ability of rGO to capture the excited state electrons from PANI-Ru at ultrafast time scales. This indicates the occurrence of electron transfer dynamics with rGO acting as an electron acceptor while PANI-Ru as a donor. Another feature significantly observed in Fig. 9b is shifting of ESA hump towards lower energy (~690 nm). This suggests that the shallow traps nearby conduction band minima are being populated enough which leads to band-gap renormalization (BGR-inhibition of bandgap) of rGO/PANI-Ru:PCBM.

Furthermore, transient decay kinetics was analyzed at two wavelengths (i.e. 560 nm and 690 nm) and the corresponding decay components are mentioned in Table 1. It is clearly observed that majority of carriers (~76%) of PANI-Ru:PCBM probed at 560 nm decayed with ~561fs while that of rGO/PANI-Ru:PCBM decayed with ~87fs (~71%). This decay lifetime has been increased to ~77fs (~71%) at 690 nm for PANI-Ru:PCBM and decreased to ~32fs (~82%) for rGO/PANI-Ru:PCBM. Since the TA measurements observed at 690 nm indicates that band gap of rGO/PANI-Ru:PCBM has been reduced, this should strengthen the electron transfer rate. To further confirm, apparent rate constants of electron transfer (K_el_) are calculated at three probe wavelengths using equation mentioned below:


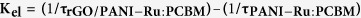


The electron transfer rate constants are calculated to be −0.187 × 10^12^ s^−1^ at 560 nm, ~0.532 × 10^12^ s^−1^ at 624 nm and ~0.86 × 10^12^ s^−1^ at 690 nm. This proves that with the decrease in bandgap, electron transfer rate is getting enhanced. The excited state carriers separation time (within few hundreds of femtoseconds) is quite faster than their recombination time (~10–100 ps), suggesting the ultrafast injection of electrons from PANI-Ru to PCBM via rGO sheets. Hence, this depicts excellent electron acceptor and transporter behavior of rGO in the metallopolymer nanocomposite.

### Graphene-metallopolymer nanocomposite as a hole transporting layer

**Figure 10:**
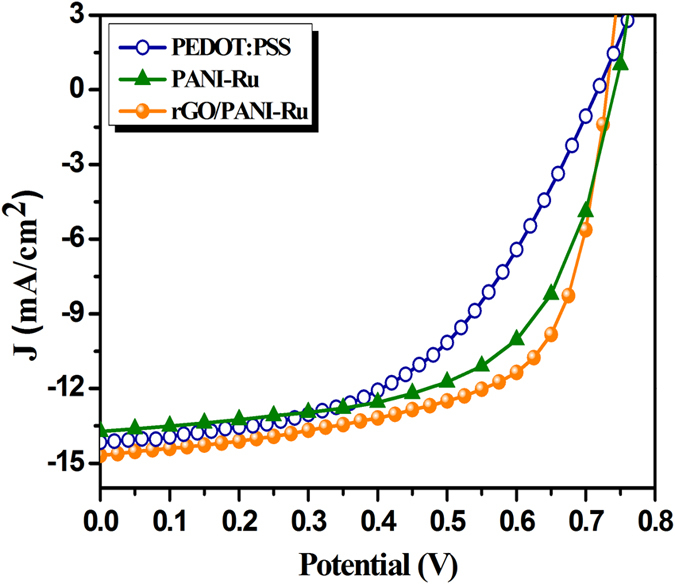
J-V characteristic curve of PTB7:PCBM used inverted PSC devices.

In addition to the electron donor, the resultant PANI-Ru and rGO/PANI-Ru nanocomposite was also successfully employed as a hole transporting layer in inverted type PSC with a device architecture of ITO/PFN/PTB7:PCBM/PANI-Ru or rGO-PANI-Ru/Al. Fig. 10 shows the J-V characteristics of inverted type PSC using PEDOT:PSS, PANI-Ru and rGO/PANI-Ru as a hole transporting layer. The PEDOT:PSS used device exhibits the PCE of 5.1%. It is interesting to note that the PANI-Ru and rGO/PANI-Ru nanocomposite as the hole transporting layer considerably enhance the PCE up to 6.1 and 6.8%, respectively (Table 3). The improvement in the photovoltaic performance of rGO/PANI-Ru nanocomposite compared to that of PEDOT:PSS and PANI-Ru is due to the superior carrier separation and charge transport properties of rGO. It is well known that the PEDOT:PSS is the most widely used hole transporting layer in PSC. However, PEDOT:PSS has several disadvantages such as strong acidic nature and hygroscopic properties, which greatly renders the performance of the solar cells^60^. In case of inverted type PSC, the polymer photoactive layer is easily degraded when high acidic PEDOT:PSS is spin coated over the photoactive layer. Therefore, rGO based metallopolymer nanocomposite may be considered as an alternative for PEDOT:PSS in inverted type PSCs.

## Conclusions

A simple wet chemical approach was adopted to synthesize metallopolymer (PANI-Ru) grafted rGO nanocomposites. The interaction between PANI-Ru and rGO sheets not only enhanced the optical absorption but also quenched the PL emission intensity. The ultrafast dissociation (ps) of excited state charge carriers in resultant hybrids were confirmed through transient absorption measurements. The PANI-Ru and rGO/PANI-Ru hybrids were used as the electron donor in bulk heterojunction polymer solar cells. The excellent electronic properties of rGO in resultant nanocomposites significantly improved the open circuit potential (*V*_*oc*_) of the device. Therefore, the rGO based light harvesting metallopolymer hybrids may find an application in opto-electronic based devices.

## Methods

### Materials

Graphite powder (synthetic, conducting grade, 325 mesh, 99.9995%) was purchased from Alfa Aesar. RuCl_3_.3H_2_O, 2(2-Pyridyl) benzimidazole, aniline monomer and aluminium (Al) evaporation slug were procured from Sigma-Aldrich. Pre-patterned ITO substrates, PEDOT:PSS, P3HT, PTB7, PC_60_BM and PC_70_BM were purchased from OSSILA, Ossila Limited, Kroto Innovation Centre, Broad Lane, Sheffield, S3 7HQ, UK. All other solvents and reagents were received and used without further purification.

### Preparation of graphene oxide (GO) and reduced graphene oxide (rGO)

GO was synthesized from graphite powder using modified Hummers method^61^. rGO was prepared using chemical reduction method using sodium borohydride as a reducing agent^62^. Briefly, 100 mg of GO was dispersed in 200 mL of DI water (0.5 mg/mL) and heated to 80–90 °C for 30 min in an oil bath under magnetic stirring. Then, 50 mL of 150 mM of NaBH_4_ was slowly added into the solution. Therefore, the color of the solution was changed from yellow to black. This indicated the successful reduction of GO to rGO. Finally, the rGO was filtered and washed several times with water, HCl and then dried in a hot air oven.

### Synthesis of polyaniline (PANI) and rGO/PANI

PANI was prepared as reported earlier^63^. In detail, 2.4 mL of aniline monomer and 47 mL of conc. H_2_SO_4_ were mixed together. Then, this mixture was dissolved in 200 mL of DI water and from that 192 mL were taken for further reaction. To this mixture 392 mg of γ-Fe_2_O_3_ was added and refluxed at 100 °C in an oil bath for 3 h under constant stirring. The obtained dark greenish PANI was cooled down to room temperature and washed several times with DI water and ethanol. Similarly, the required amount of rGO was added during the synthesis of PANI to obtain rGO/PANI composite.

### Preparation of Ruthenium complex

The ruthenium complex was prepared as per the previous report^64^. In detail, a mixture of 2-(2-pyridyl) benzoimidazole (0.09 g, 0.48 mmol) and RuCl_3_·3H_2_O (0.10 g, 0.48 mmol) was refluxed in 15 mL of absolute ethanol for 4 h. Then the reaction mixture was cooled down to room temperature. Finally, a brown precipitate was collected by filtration, washed with excess ethanol and dried to afford an analytically pure {2-(2-pyridyl) benzimidazole}RuCl_3_ complex as a brown solid. The schematic representation for the preparation of Ru complex is given in Fig. S6 (Supplementary Information).

### Synthesis of PANI-Ru and rGO/PANI-Ru nanocomposites

RuCl_3_· 3H_2_O (12.5 mg) and 2(2-Pyridyl) benzimidazole (11 mg) were dissolved in 15 mL of ethanol and refluxed for 4 h. After 15 min of reflux started, the required amount of PANI was added to obtain PANI-Ru. Similarly, the rGO/PANI was added under identical experimental condition to obtain rGO/PANI-Ru hybrid nanocomposites.

### Characterization studies

UV-visible absorption spectra were recorded using Specord 200-plus (Analytikjena, Germany). The morphology for the prepared samples and the thickness of the photoactive layer was analyzed by FEI Quanta FEG 200 HR-SEM. TEM images were obtained through A JEM-2100 JEOL (Japan) with an accelerating voltage of 120 kV. The chemical oxidation states of the elements were analyzed by X-ray photoelectron spectra recorded through a Kratos Axis-Ultra DLD spectrometer with Mg-Kα radiation. Fourier Transform Infrared (FT-IR) spectra for the samples were monitored by Agilent, Cary 660, USA. The grafting of polyaniline onto rGO sheets were confirmed by Raman spectra using Raman spectrophotometer (Nanophoton Raman 11, Japan). AFM images of photoactive films were obtained from Atomic Force Microscope (AFM) (Multimode V (NS-V) Veeco, US). The Photoluminescence emission spectra were recorded from Horiba Fluorolog@3 instrument. Ultrafast spectroscopy measurements were performed using Helios ultrafast spectroscopic system, which comprising of an oscillator (Micra from coherent), amplifier (Legend from Coherent) and OPA (from light conversion). The output (4 mJ, 1 kHz) of the amplifier with the pulse width of 35 fs centred at 800 nm is splitted into two parts. The OPA is fed with 1.8 mJ energy while 0.5 mJ is fed to the spectrometer via a delay stage of 0–8 ns. The splitted beam passed through the sapphire plate generated the broad band white light inside the spectrometer, which is capable of generating the probe pulse of 350–850 nm. A 480 nm of the OPA output was selected as pump beam with a fluence of 20 μJ/cm^2^. The surface explorer software was used for the analysis and fitting.

### Photoelectrochemical studies

Photoelectrochemical measurements were performed using an electrochemical workstation (CHI608E) with three electrode configuration (Pt-wire as counter electrode, Ag/AgCl as reference electrode and hybrid nanocomposites coated on FTO as a working electrode). The 0.1 M Na_2_SO_4_ solution was used as an electrolyte and a Xe lamp (250 W, OSRAM, Germany) was used as a light source for irradiation. The working electrode was prepared as reported earlier^19^. In brief, 50 mg of PANI-Ru or rGO/PANI-Ru nanocomposite was added with 150 μL of PEG (mol. wt 400) and 125 μL of ethanol to make slurry. Finally, the prepared slurry was uniformly coated on a 2.5 × 2.5 cm^2^ fluorine-doped tin oxide (FTO) glass substrate by doctor blade technique using scotch tape as a spacer. The active area of the electrode was about 1 × 1 cm^2^. Prior to the analysis, the electrode was dried in an oven for 45 min.

### Electrochemical studies

To calculate the HOMO and LUMO energy levels of resultant nanocomposite, cyclic voltammetry (CV) studies was performed as reported earlier^57^ using a typical three electrode assembly consists of Pt wire, Ag/AgCl and rGO/PANI-Ru modified glassy carbon as a counter, reference and working electrode, respectively. A 0.1 M NaCl aqueous solution was used as an electrolyte. The catalyst slurry was prepared by adding rGO/PANI-Ru nanocomposite in a DMF solution and ultrasonicated for 30 min. Then, the catalyst ink was carefully dropped on the well polished glassy carbon surface and dried for overnight. Prior to the CV analysis, the electrolyte solution was purged with N_2_ for 15 min. Finally, the CV measurement was carried out in the potential range of −1 to +1 V at the scan rate of 100 mV/s.

### Photovoltaic device fabrication and J-V measurements

Pre-patterned ITO coated glass substrates (20 Ω/sq) were cleaned with soap solution and followed by ultrasonication in acetone, DI water and isopropyl alcohol for 20 min each. Then, the cleaned substrates were dried in an oven and exposed with UV ozone for 20 min. For PANI based PSCs, a thin layer of PEDOT:PSS was spin coated on cleaned ITO substrate at 3000 rpm for 1 min and annealed at 140 °C for 10 min. After that a photoactive blend of PANI-Ru or rGO/PANI-Ru:PCBM (1:4) in DMF:o-dichlorobenzene (1:1) was spin coated over PEDOT:PSS layer at 1000 rpm for 1 min followed by annealing at 150 °C for 15 min in N_2_ atmosphere. For PANI based hole transport layer, PFN was spin coated on cleaned ITO substrate at 3000 rpm for 1 min and annealed at 200 °C for 10 min^65^ and after that PTB7:PCBM in chlorobenzene were spin coated at 1000 rpm for 1 min followed by annealing at 80 °C for 15 min in N_2_ atmosphere. After that, PANI-Ru or rGO/PANI-Ru were spin coated on the top of active layer. Finally, a 100 nm Al layer was deposited on active area using thermal evaporator under a vacuum of 10^−6^ Torr. The active area of the device was 6 mm^2^. The final device architecture consist of ITO/PEDOT:PSS (~30 nm)/PANI-Ru or rGO/PANI-Ru:PCBM (~300 nm)/Al (100 nm) and ITO/ZnO/PTB7:PCBM/PANI-Ru or rGO/PANI-Ru (~30 nm)/Al (100 nm). Current density-voltage (J-V) characteristics of the fabricated PSC device were measured inside of glove box using AM 1.5 G solar simulator with 100 mW/cm^2^ output (Oriel sol 3ATM solar simulator and Keithley 2420-C 3 A source meter).

## Additional Information

**How to cite this article:** Vinoth, R. *et al*. Ruthenium based metallopolymer grafted reduced graphene oxide as a new hybrid solar light harvester in polymer solar cells. *Sci. Rep.* **7**, 43133; doi: 10.1038/srep43133 (2017).

**Publisher's note:** Springer Nature remains neutral with regard to jurisdictional claims in published maps and institutional affiliations.

## Supplementary Material

Supplementary Information

## Figures and Tables

**Table 1:** Decay kinetics of transient absorption for PANI-Ru:PCBM and rGO/PANI-Ru:PCBM at 560 and 690 nm probe wavelengths.

Photoactive Film	λ (nm)	A_1_	τ_1_ (ps)	A_2_	τ_2_ (ps)	A_3_	τ_3_ (ps)
**PANI-Ru:PCBM**	560	0.765	0.561	0.11	11.7	0.0558	473
	690	0.712	0.775	0.144	14.4	0.0565	274
**rGO/PANI-Ru:PCBM**	560	0.711	0.87	0.147	13.9	0.0561	283
	690	0.836	0.319	0.0864	5.92	0.0442	125

**Table 2:** Photovoltaic parameters such as ***J***_***sc***_ ***, V***_***oc***_ ***, FF*** and ***η*** of the PSC device fabricated with PANI-Ru and rGO/PANI-Ru as an electron donor.

*p*-type	*J*_*sc*_ (*mA*/*cm*^*2*^)	*V*_*oc*_ (*V*)	*FF*	*η* (average) × % (10^−3^)
PANI-Ru	0.014	0.03	0.23	0.10 (0.09)
rGO/PANI-Ru	0.030	0.18	0.27	1.45 (1.39)

Average η was calculated based on the data of PSC devices (4 Nos) fabricated under same experimental conditions.

**Table 3:** Photovoltaic parameters such as ***J***_***sc***_ ***, V***_***oc***_ ***, FF*** and ***η*** of the inverted PSC devices fabricated with different hole transporting layers.

*Hole Transporting Layer*	*J*_*sc*_ (*mA/cm*^*2*^)	*V*_*oc*_ (*V*)	*FF*	*η* (%) average
PEDOT:PSS	14.16	0.717	0.503	5.1 (4.9)
PANI-Ru	13.72	0.741	0.600	6.1 (5.8)
rGO/PANI-Ru	14.59	0.732	0.636	6.8 (6.6)
