# Supplementary material for: Ruthenium based metallopolymer grafted reduced graphene oxide as a new hybrid solar light harvester in polymer solar cells
Source: Sci Rep. 2017 Feb 22;7:43133. doi: 10.1038/srep43133 (PMC5320515; doi:10.1038/srep43133)
Supplement: Supplementary Information [file srep43133-s1.doc]

**Supplementary information**

**Ruthenium based metallopolymer grafted reduced graphene oxide as a new hybrid solar light harvester in polymer solar cells**

R. Vinoth^1^, S. Ganesh Babu^1^, Vishal Bharti^2^, S. Venkataprasad Bhat^1^, C. Muthamizhchelvan^3^, Praveen C Ramamurthy^4^, Vinay Gupta^2,*^, Chhavi Sharma^5^, Dinesh K Aswal^6^, M. Navaneethan^7^, Yasuhiro Hayakawa^7^ & B. Neppolian ^1,*^

**^1,*^**SRM Research Institute, SRM University, Kattankulathur, Kancheepuram 603203 (D.t.), Tamil Nadu, India. **^2,*^**Organic and Hybrid Solar Cell Group, National Physical Laboratory, Dr. K. S. Krishnan Marg, New Delhi 110012, India. **^3^**Center for Materials Science and Nano Devices, Department of Physics, SRM University, Kattankulathur, Kancheepuram 603203 (D.t.), Tamil Nadu, India. **^4^**Department of Materials Engineering, Indian Institute of Science, Bangalore 560012, India. **^5^**Ultrafast Optoelectronics and Terahertz Photonics group, Physics of Energy Harvesting Division CSIR-National Physical Laboratory, New Delhi- 110012, India. **^6^** National Physical Laboratory, Dr. K. S. Krishnan Marg, New Delhi 110012, India. **^7^**Research Institute of Electronics, Shizuoka University, 3-5-1 Johoku, Naka-Ku, Hamamatsu, Shizuoka 432-8011, Japan. Correspondence and requests for materials should be addressed to V.G. (email: [drvinaygupta@netscape.net](mailto:drvinaygupta@netscape.net)) or B.N. (email: neppolian.b@res.srmuniv.ac.in)

**Figure S1.** FTIR spectra of PANI and Ru complex

**
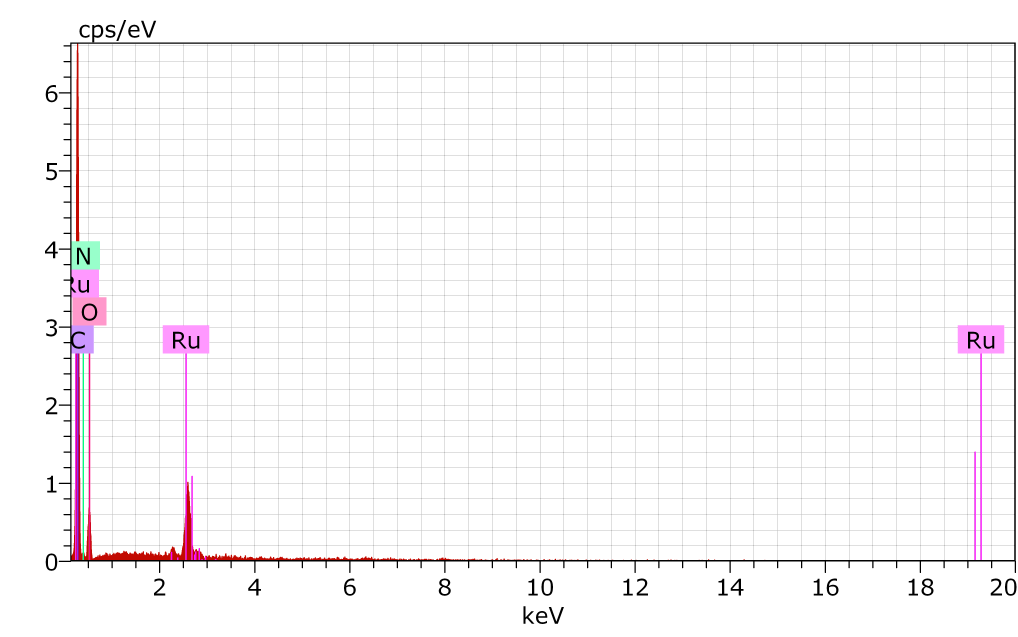
**

**Figure S2.** EDX spectra of rGO/PANI-Ru nanocomposites.


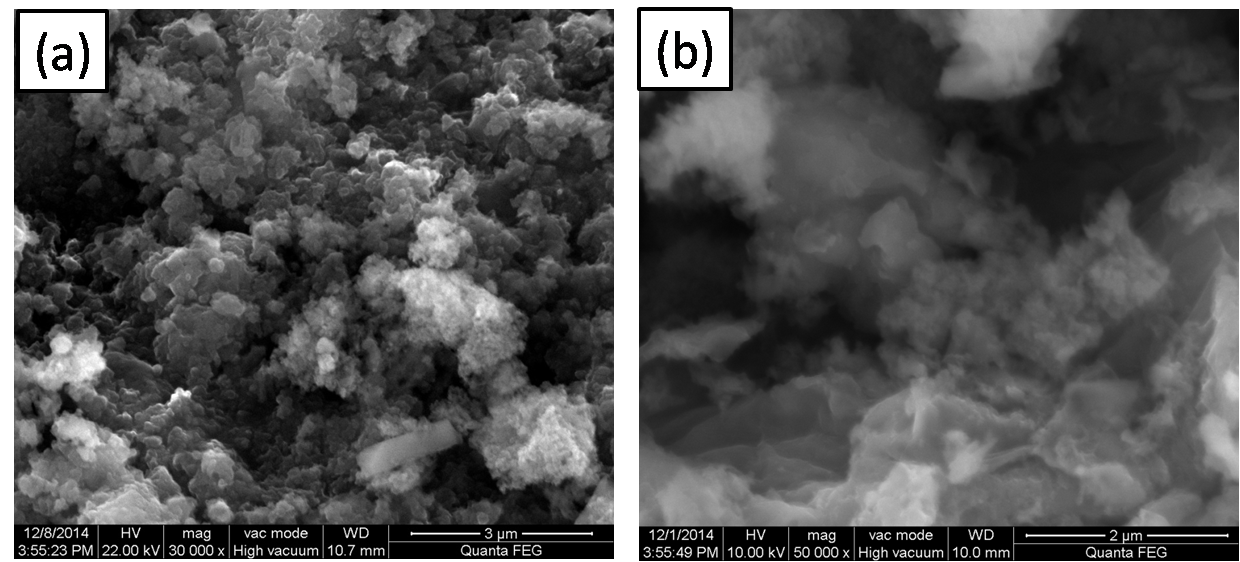


**Figure S3.** FE-SEM image of PANI and rGO/PANI.


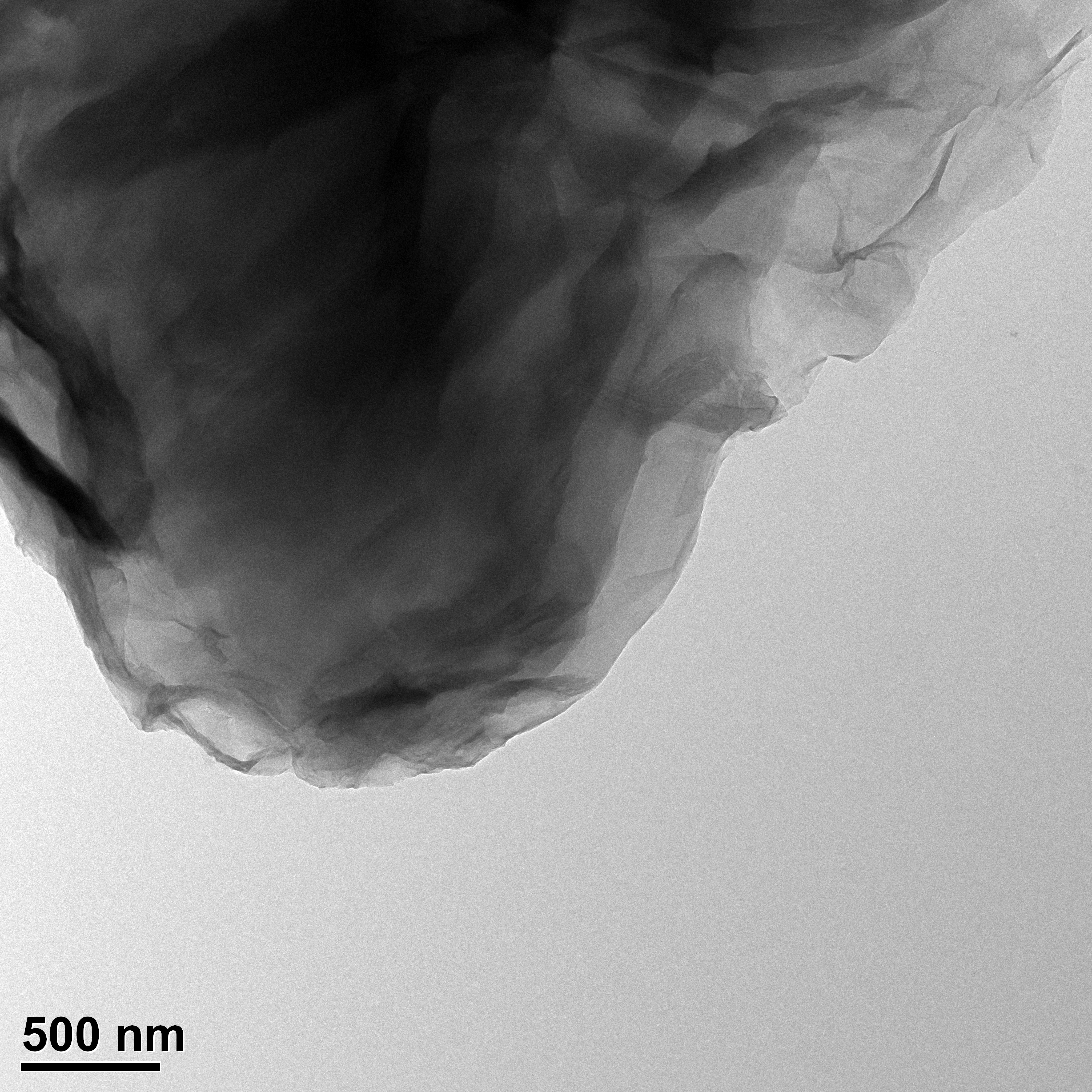


**Figure S4.** TEM image of rGO.

**
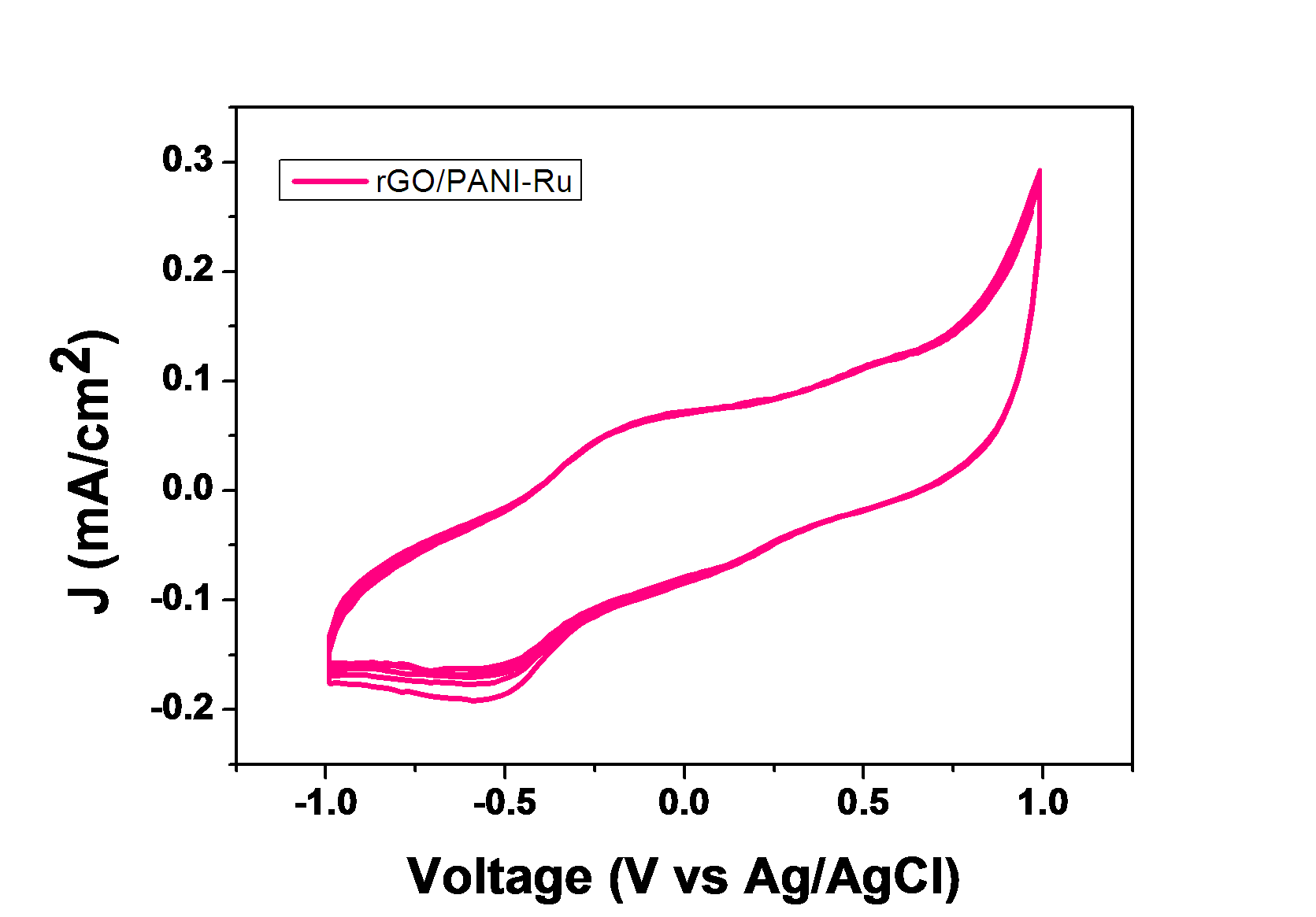
**

**Figure S5.** Cyclic Voltammagram (CV) curve of rGO/PANI-Ru coated on glassy carbon in 0.1M NaCl at the scan rate of 100 mV/s.

**
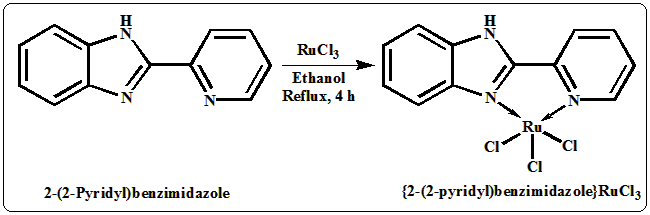
**

**Figure S6.** Schematic representation for the preparation of {2-(2-pyridyl)benzimidazole}RuCl_3_ complex.


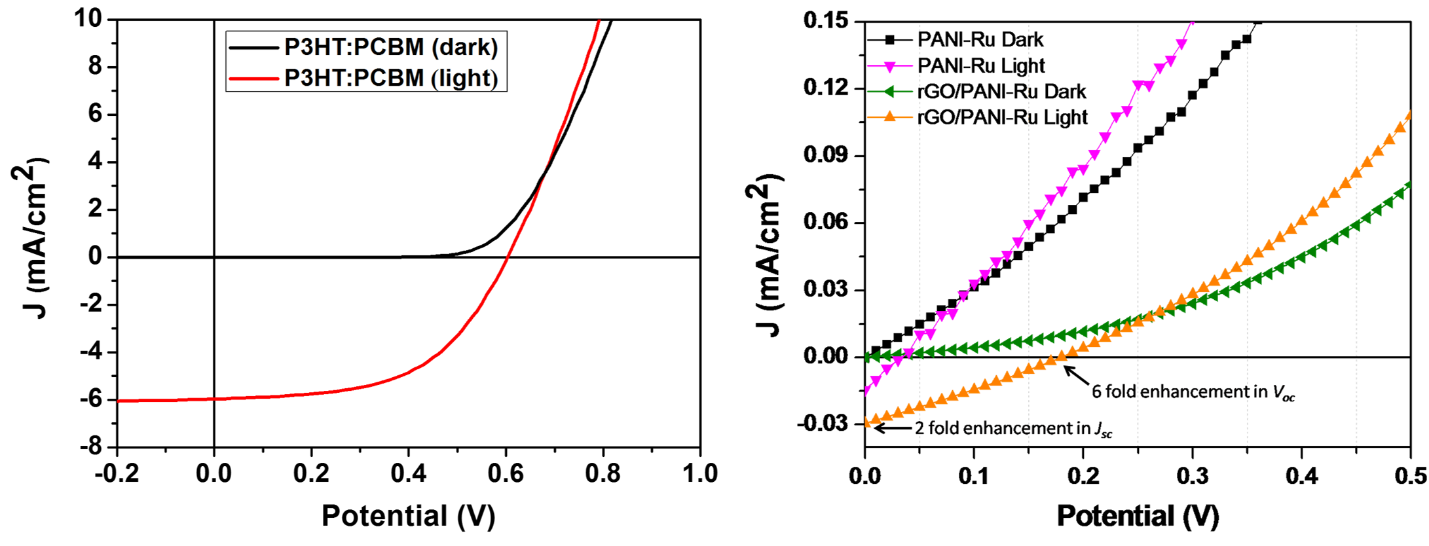


**Figure S7.** J-V characteristic curve of P3HT:PCBM used PSC device
